# Hierarchical transitions and fractal wrinkling drive bacterial pellicle morphogenesis

**DOI:** 10.1073/pnas.2023504118

**Published:** 2021-05-10

**Authors:** Boyang Qin, Chenyi Fei, Bruce Wang, Howard A. Stone, Ned S. Wingreen, Bonnie L. Bassler

**Affiliations:** ^a^Department of Molecular Biology, Princeton University, Princeton, NJ 08544;; ^b^Department of Mechanical and Aerospace Engineering, Princeton University, Princeton, NJ 08544;; ^c^Lewis–Sigler Institute for Integrative Genomics, Princeton University, Princeton, NJ 08544;; ^d^Howard Hughes Medical Institute, Chevy Chase, MD 20815

**Keywords:** bacterial pellicle, morphogenesis, *Vibrio cholerae*, soft material

## Abstract

Bacterial cells can self-organize into structured communities at fluid–fluid interfaces. These soft, living materials composed of cells and extracellular matrix are called pellicles. Cells residing in pellicles garner group-level survival advantages such as increased antibiotic resistance. The dynamics of pellicle formation and, more generally, how complex morphologies arise from active biomaterials confined at interfaces are not well understood. Here, using *Vibrio cholerae* as our model organism, a custom-built adaptive stereo microscope, fluorescence imaging, mechanical theory, and simulations, we report a fractal wrinkling morphogenesis program that differs radically from the well-known coalescence of wrinkles into folds that occurs in passive thin films at fluid–fluid interfaces. Four stages occur: growth of founding colonies, onset of primary wrinkles, development of secondary curved ridge instabilities, and finally the emergence of a cascade of finer structures with fractal-like scaling in wavelength. The time evolution of pellicle formation depends on the initial heterogeneity of the film microstructure. Changing the starting bacterial seeding density produces three variations in the sequence of morphogenic stages, which we term the bypass, crystalline, and incomplete modes. Despite these global architectural transitions, individual microcolonies remain spatially segregated, and thus, the community maintains spatial and genetic heterogeneity. Our results suggest that the memory of the original microstructure is critical in setting the morphogenic dynamics of a pellicle as an active biomaterial.

## Significance

Multicellular bacterial communities called biofilms and pellicles significantly influence medical infections and industrial biofouling. Biofilms and pellicles often act as reservoirs of toxigenic bacteria. Here, we report a fractal wrinkling morphogenesis program that underlies pellicle formation in the model biofilm former and global pathogen, *Vibrio cholerae*. Pellicle morphogenesis is marked by the emergence of a cascade of self-similar structures with fractal-like scaling in wavelength, which increases surface area and presumably enhances nutrient transport and signaling among community members. This morphogenesis program can be altered by varying the spatial heterogeneity of the community. Thus, bacterial pellicles could provide tractable model systems to understand overarching principles driving morphogenesis and for engineering of functional soft biomaterials.

Spontaneous folding, wrinkling, and curling of soft tissues and active materials are ubiquitous in nature. For example, during the development of mammalian organs and plant leaves, collections of many cells self-organize into ordered morphological structures far larger than the size of the individual cells. Nontrivial geometric patterns and architectures often link form to function, such as the fractal branching and size scaling of leaf veins (1), the gyrification of the cerebral cortex (2–4), and the curving of villi in the gut (5, 6). Bacterial cells can also self-organize into communities known as biofilms and pellicles at interfaces. These multicellular communities are crucial in contexts such as medical infections and industrial biofouling because cells in biofilms and pellicles display enhanced resilience to antibiotics, immune clearance, and physical perturbations compared to their isogenic planktonic counterparts (7–9). Analogous to eukaryotic systems, bacterial biofilms and pellicles develop striking macroscopic morphologies including wrinkles and delaminations that are driven by combined biological, material-physics, and mechanical determinants (10–14). Studies of biofilms at the single-cell level show the emergence of internal cell ordering (15–17) and collective cellular flow (18). However, the dynamics of self-organization and the sequence of mechanical instabilities that direct morphogenesis for expanding active soft materials at fluid–fluid interfaces, such as bacterial pellicles, are undefined. Critically, overarching principles connecting microscopic cell organization to macroscopic structures, while potentially common to morphogenesis across different biological systems, are not well understood.

Here, we report the sequence of architectural transitions that occur during pellicle maturation for the pathogen *Vibrio cholerae*. We discover a morphogenic transition characterized by a cascade of wrinkles with fractal scaling in wavelength, which is distinct from the classic wrinkle-to-fold transition widely observed in physical systems such as thin polymer films on fluid baths (19), nanoparticle thin sheets (20), and thin epitaxial layers of gold and elastomers (21, 22). In classic wrinkle-to-fold transitions, as compression increases beyond the linear regime, the initial wrinkles coalesce and localize into folds with large amplitudes. During such transitions, the shape evolution of passive films can be characterized by the minimization of the system’s total energy for different conformations (20, 23–25). In particular, elastic bending energies contained in small wavelength undulations are lost in favor of energies associated with large-scale folds. By contrast, as we demonstrate below, the opposite length-scale progression occurs for actively growing *V*. *cholerae* pellicles, in which finer wrinkles and creases continue to emerge following initial morphogenic transitions. The length scales of wrinkles in a mature *V*. *cholerae* pellicle follow fractal order, achieving a pattern with fractal dimension of ∼1.6. The additional dimensionality conferred by the fractal geometry could promote nutrient access and enhance signal transduction compared to a smooth structure (26). The fractal progression toward small length scales, as opposed to coalescence toward larger folds, stems from heterogeneous growth in the initial film. We find that the original distribution of microcolonies is preserved during pellicle architectural transitions, and, importantly, it determines the exact sequence of morphogenesis events that will occur. Indeed, changing the initial colony seeding density enabled us to identify a total of four morphogenic routes, which we termed the standard, bypass, crystalline, and incomplete modes. Our results demonstrate a direct connection between microscopic structure and macroscopic morphology for an active, growing soft biomaterial.

## Hierarchical Morphogenic Transitions and Fractal-Order Wrinkling Direct the Standard Mode of *V*. *cholerae* Pellicle Formation at a Fluid–Fluid Interface

**Fig. 1.**
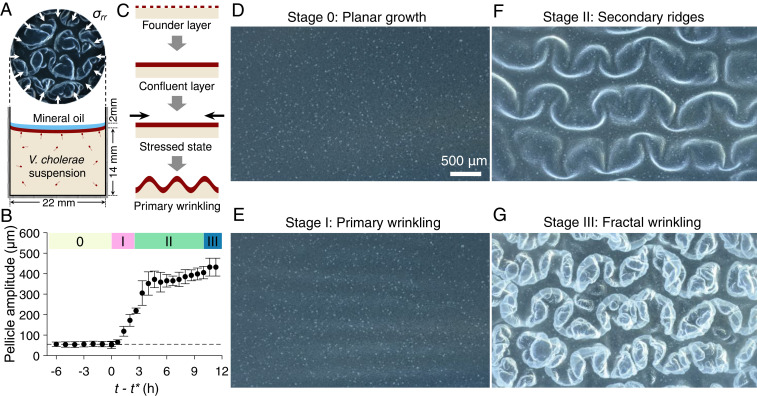
*V*. *cholerae* pellicles undergo hierarchical transitions from smooth 2D films to folded surfaces with fractal scaling in wavelength. (*A*) Schematic of the growth chamber used to assay *V*. *cholerae* pellicle formation at a liquid–liquid interface. A thin layer of sterile mineral oil (blue) covers the bacterial pellicle (dark red). The chamber walls confine the confluent pellicle via radial stresses *σ*_*rr*_ (depicted by arrows). (*B*) Time development of pellicle deformation amplitude in the out-of-plane direction, defined as the vertical distance between the top-most and bottom-most regions of the pellicle layer (*n* = 3 biological replicates). Cell density at inoculation: OD_600_ = 0.01. The data show the change in pellicle wrinkle amplitude versus time *t*, offset by the onset time of primary wrinkling t*, a proxy for compressive stress due to pellicle expansion. We note that t* is ∼26 h, and the pellicle max–min amplitude does not increase appreciably prior to t*. The four morphogenic stages are labeled 0, I, II, and III, respectively. Error bars denote SEs. (*C*) Schematic of the early stages of bacterial pellicle formation. In the first row, pellicle formation initiates with founder microcolonies at the fluid–fluid interface. In the second row, pellicle surface coverage increases to confluency. In the third row, compressive stress (black arrows) develops within the pellicle and drives the onset of a buckling instability and primary wrinkling (fourth row). (*D*–*G*) Pellicle top views of the four morphogenic stages in the standard mode as labeled in *B*. The sequential stages are as follows: (*D*) Stage 0, 2D planar growth; (*E*) Stage I, onset of primary wrinkling; (*F*) Stage II, emergence of curved secondary ridge instabilities in the direction transverse to the primary wrinkles; and (*G*) Stage III, development of increasingly finer structures and fractal-like wrinkling. Images in *D*–*G* were acquired with a custom-built stereo microscope using focus-stacking algorithms of volumetric scans in the vertical direction.

We begin by imaging the sequence of *V*. *cholerae* pellicle morphogenic transformations that occur for initial cell seeding densities from OD_600_ = 0.005 to 0.1 in lysogeny broth (LB) medium. We will refer to this set of events as the standard mode of pellicle formation. Using a custom stereoscope setup that adaptively tracked pellicle features in the test wells, we acquired continuous volumetric scans of *V*. *cholerae* pellicles as they formed at the interface between the growth medium and mineral oil (*Materials and Methods*, Fig. 1*A*, and Movie S1). In studies of embryonic development, application of a thin layer of sterile mineral oil is a well-established nontoxic method to allow gas exchange while minimizing evaporation and vibrations (27–29). Using this strategy allowed us to monitor pellicle morphogenesis from the early microcolony stage through maturation (6 h to 48 h, Movie S2). Our setup also enabled measurement of the amplitudes of pellicle deformations that occurred in the out-of-plane (vertical) direction (Fig. 1*B* and *SI Appendix*, Fig. S1). *V*. *cholerae* pellicle development begins with microcolony formation initiated by a founder cell layer (Fig. 1*C*, top row) that extends in two-dimensions at the fluid–fluid interface (Fig. 1*D*). Once the planar pellicle reaches confluence (Fig. 1*C*, second and third rows), the layer buckles and formation of primary wrinkles occurs (Fig. 1*C*, bottom row and Stage I, Fig. 1*E*). Next, curved ridges form in the direction transverse to the primary wrinkles (Stage II, Fig. 1*F*). In the final stage, wrinkles of smaller wavelength continue to develop along the secondary ridges, forming an irregular cascade of progressively finer structures (Stage III, Fig. 1*G*). This sequence of transformations presumably relieves accumulated strain energy via formation of finer wrinkles and moreover is grossly analogous to a vortex cascade in turbulence in which kinetic energy is transferred from larger to increasingly smaller length scales (30, 31).

## Pellicle Morphogenesis Begins with Microcolony Growth and the Onset of Primary Wrinkling Instabilities

**Fig. 2.**
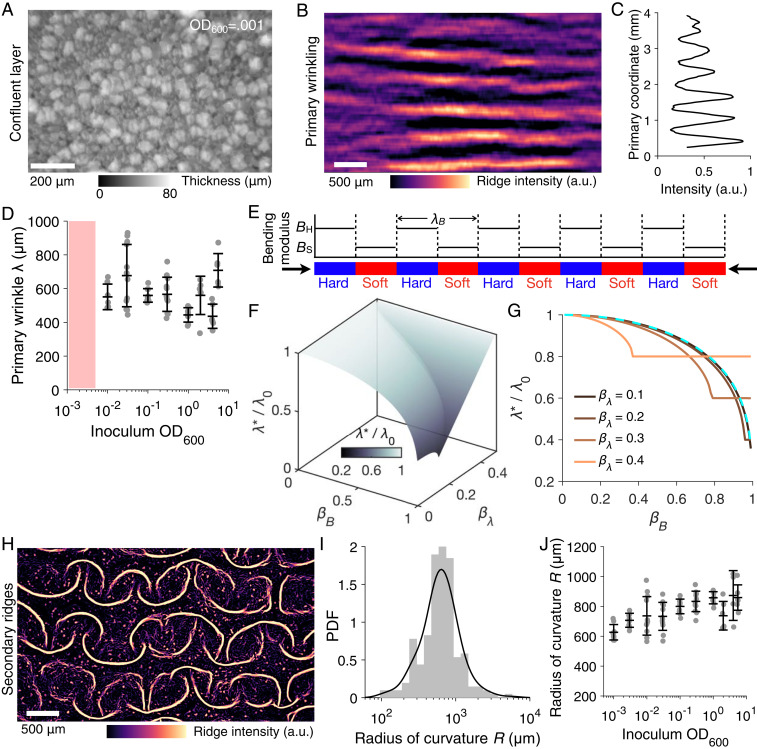
Primary wrinkling and subsequent secondary ridge instabilities drive morphogenesis of *V*. *cholerae* pellicles. (*A*) Pellicle thickness heterogeneity prior to the onset of the primary wrinkling instability for initial cell seeding density of OD_600_ = 0.001. Intensities denote local relative thickness of the pellicle obtained from reflectance imaging calibrated with vertical *z*-scanning. (*B*) The pellicle primary wrinkling profile was measured as ridge (Frangi vesselness) intensities of the wrinkles. (*C*) The intensity profile variation extracted from *B*, from which the dominant wavelength can be obtained. (*D*) The primary wrinkling wavelength *λ* measured for pellicles obtained from different initial inoculum seeding densities. The hatched red region denotes the bypass mode in which primary wrinkling does not occur, but rather, the film transforms directly from Stage 0 to Stage II (Fig. 1). Data points show biological replicates for technical triplicates. (*E*) Schematic of the modeled heterogeneous pellicle with alternating regions of high (blue, *B*_H_) and low (red, *B*_S_) bending moduli. The wavelength of the alternating pattern is *λ*_*B*_. The pellicle heterogeneity can be characterized by two dimensionless parameters: the modulus ratio *β*_*B*_ and the length scale ratio *β*_*λ*_. (*F*) Prediction from the analytical model for the primary wrinkling wavelength for a thin film possessing heterogeneous bending moduli. The colored surface indicates the possible set of wrinkling wavelengths in the parameter *β*_*B*_ and *β*_*λ*_. Here, λ* is the wrinkle wavelength of the heterogeneous film and *λ*_0_ is the wrinkle wavelength of a smooth film with a uniform bending modulus. (*G*) Representative cuts through the isosurface in *F*. Colors denote *β*_*λ*_ values. The dashed cyan curve represents the wrinkling wavelength predictions from harmonic averaging of the pellicle thickness. (*H*) The pellicle surface profile during Stage II (secondary folding, Fig. 1*F*) as measured by ridge (Frangi vesselness) intensity. (*I*) The probability density function (PDF) of the radius of curvature *R* for the segmented secondary ridges in Stage II in *H*. The mean radius of curvature is <*R>* = 730 μm. (*J*) The average radius of curvature of the secondary folding *R* is shown for the specified initial inoculum seeding densities. Data points show biological replicates for technical triplicates.

*V*. *cholerae* pellicle development, as documented above, begins with microcolony formation, presumably initiated by single founder cells that adsorb to the fluid–fluid interface and grow as isolated entities to 10 to 50 μm in diameter before a thinner second layer of cells expands and fills the microcolony-free regions at the interface (*SI Appendix*, Fig. S2). The number density and size distributions of the microcolonies varied with the initial cell seeding density (*SI Appendix*, Fig. S3). Irrespective of the cell seeding density, the pellicle at the planar film stage was not smooth but rather harbored regions of varying thicknesses that were embedded with microcolonies (Fig. 2*A* and *SI Appendix*, Fig. S2). Once the pellicle expanded into a confluent layer in contact with the well boundary, the buildup of compressive stress triggered the first morphologic transition from a two-dimensional (2D) planar sheet to a wrinkled surface along one apparently random direction (Fig. 2 *B* and *C*), presumably the direction under the largest stress, similar to the classic wrinkling instability of smooth thin films (21, 32, 33) and modeled below. For our conditions, the wrinkle wavelength was ∼600 μm and was independent of the initial seeding density in the range where the standard mode occurred (OD_600_ = 0.005 to 0.1, Fig. 2*D*).

## Mathematical Modeling Reveals that the Pellicle Biomaterial Properties and Its Heterogeneous Microstructures Drive the Emerging Wrinkle Wavelength

How does the presence of microcolonies of order 10 μm in size and the resulting heterogeneity in film thickness affect the pellicle wrinkle wavelength? To understand the emergent wavelength, we developed a simplified one-dimensional (1D) wrinkling model that incorporates the heterogeneous film structure by engendering local differences in pellicle bending stiffness. We treat the pellicle as a thin elastic film with alternating high (*B*_H_) and low (*B*_S_) bending moduli with a spatial wavelength of *λ*_*Β*_ (Fig. 2*E*). Two dimensionless parameters describe the pellicle surface heterogeneity, the relative stiffness difference *β*_*B*_ = (*B*_H_−*B*_S_)/(*B*_H_*+B*_S_) and the relative pattern wavelength *β*_*λ*_ = *λ*_*Β*_*k*_0_/2π, where *k*_0_ denotes the wrinkling wavenumber of a uniform film with an equivalent average modulus (*Materials and Methods*). We note that local differences in pellicle bending stiffness can be related to local thickness *h,* according to *B*∼*h*^3^ from linear elasticity theory. The resulting wrinkling profile and wavelength are obtained by solving the Föppl–von Kármán equation. Notably, compared to a homogeneous film of the same average stiffness, our model shows that the wrinkle wavelength of a heterogeneous film decreases with increasing surface modulus heterogeneity *β*_*B*_ (Fig. 2 *F* and *G*). Our model predicts two wrinkling regimes depending on the amplitude *β*_*B*_ and the wavelength *β*_*λ*_ of the heterogeneity pattern: for small values of *β*_*B*_ and *β*_*λ*_, the wrinkle profile is locally modified from that of a homogeneous smooth film (*SI Appendix*, Fig. S4*A*); for larger *β*_*B*_ and *β*_*λ*_, the highly curved peaks and valleys are confined to the soft portions, while the stiff portions remain essentially without curvature, resulting in a constant wavelength that is twice the prescribed modulus wavelength *λ*_*B*_ (*SI Appendix*, Fig. S4*B*). The model also predicts that, compared to a smooth film, the heterogenous film is more compliant and thus can undergo larger deformations prior to wrinkling (*SI Appendix*, Fig. S4 *C* and *D*). Furthermore, finite element simulations of a pellicle with sinusoidal variations in local film thickness and bending modulus showed quantitative agreement with the analytical theory (*SI Appendix*, Fig. S5). Thus, the heterogeneous microstructure of the pellicle, which is tied to thickness variations, is important for driving the initial mechanical instability and, in turn, dictating the primary wrinkle wavelength.

## The Initiation of Secondary Ridge Instabilities Sets the Global Pellicle Morphology

Our next goal was to follow the morphological transitions that occur in the pellicle subsequent to wrinkle formation. The 1D primary wrinkles are transient and rapidly progress into an ordered 2D pattern via a secondary mechanical instability. Curved ridges emerge along the direction orthogonal to the primary wrinkles in a pattern of interlocked arcs (Fig. 2*H*). Specifically, the primary wrinkles bend in the transverse direction and undergo a 1D-to-2D transition. These secondary ridge structures are initially disjointed (Stage II, Fig. 1*F*) with a mean radius of curvature around 730 μm (Fig. 2*I*), similar to the primary wrinkle wavelength. The average radius of curvature modestly increased at higher initial cell seeding density (Fig. 2*J*). This finding suggests that a common length scale, potentially determined by the pellicle material properties and microstructures (Fig. 2 *F* and *G*), sets the first two stages of morphogenesis.

## A Cascade of Fractal Wrinkles Marks *V*. *cholerae* Pellicle Maturation

We wondered whether the 2D pellicle ridges that formed (Figs. 1*F* and 2*H*) would further increase in amplitude and merge into folds, as expected for passive thin films at fluid–fluid interfaces (20, 23, 25) and for biofilms on agar surfaces (10). In smooth thin elastic films floating at liquid–air interfaces, the wrinkle-to-fold transition relieves the total energy accumulated in the film, consisting of liquid potential energy and bending energy. This transition is marked by the coalescence of spatially uniform wrinkles into localized upward and downward pointing folds or S folds (20).

**Fig. 3.**
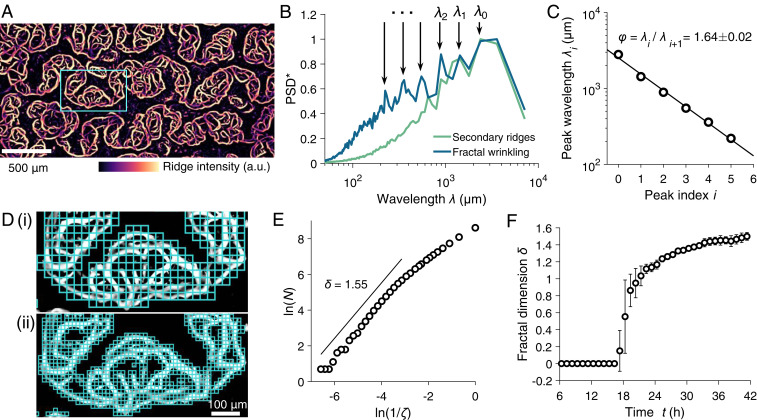
*V*. *cholerae* pellicle maturation exhibits the emergence of features with progressively smaller wavelengths and development of fractal wrinkles. (*A*) Fractal wrinkling of the *V*. *cholerae* pellicle as measured by ridge intensities at an inoculum seeding density of OD_600_ = 0.01. (*B*) Normalized power spectral density (PSD*) of ridge intensities in the direction orthogonal to the primary wrinkling for both Stage II secondary ridges (green) and Stage III fractal wrinkling (blue). The discrete spectral peaks that occur at progressively smaller wavelengths are labeled *λ*_*i*_ with *i* = 0, 1, and 2. (*C*) The spectral peak wavelengths from *B* as a function of peak index *i*. The ratio of consecutive peak wavelengths is denoted *φ*. (*D*) The fractal morphology of the pellicle measured using a box-counting scheme at increasingly finer length scales *ζ*, in i and ii, respectively. The skeletonized pellicle ridge intensities in the highlighted box in *A* were used. (*E*) The Hausdorff dimension *δ* = 1.55 due to the wrinkled pellicle morphology was extracted from the scaling of box count *N* versus box length scale *ζ* for the images shown in *D*. (*F*) The time evolution of the fractal dimension *δ* through the complete *V*. *cholerae* pellicle morphogenesis transition (Stages 0 through III).

We observed a radically different developmental transition for the pellicle, an actively growing biofilm consisting of heterogeneous bacterial colonies. Pellicles deform both elastically and plastically as bacterial cells divide and secrete matrix. Moreover, pellicles are far from smooth; they possess significant microstructural features. As the pellicle expanded, wrinkles of smaller and smaller wavelength and progressively finer structures emerged along the secondary ridges (Figs. 1*G* and 3*A*). Analysis of the power spectra in the direction orthogonal to the primary wrinkles shows the excitation of a cascade of wrinkle wavelengths (Fig. 3*B*). These wrinkles are self-similar, with a decay in the consecutive peak wavelengths *φ = λ*_*i*_/*λ*_*i*+1_ following a ratio of *φ* = 1.64 (Fig. 3*C*), in stark contrast to the increase in wavelength (period doubling) that occurs in passive smooth films. We note the ratio *φ* is close to the golden ratio *ϕ =* 1.62 associated with the ratio of consecutive Fibonacci numbers. Defined as a sequence that grows logarithmically, Fibonacci numbers are found in the phyllotactic ordering of leaves in plants that arise as a consequence of iterative self-organization (34) and in the spiral patterns of self-assembled spherules that decorate microshells and minimize total strain energy (35).

Self-similarity strongly suggests fractal behavior. Indeed, estimation by box-counting algorithms of the skeletonized pellicle wrinkles (Fig. 3*D* and *Materials and Methods*) demonstrates a fractal dimension of *δ* = 1.55, as defined by a power–law relationship of over four scale orders (Fig. 3*E*). Quantitation of *δ* in time shows that as the *V*. *cholerae* pellicle features transition from smooth ridge structures to mature fractal wrinkles, the Hausdorff dimension increased from 1 to around 1.6 (Fig. 3*F*). A cascade of wrinkling has also been previously reported for *Bacillus subtilis* pellicles near the region in contact with the vertical wall due to edge and capillary effects (13), similar to the edge fractal wrinkle of thin sheets (36). Additional dimensionality increases the effective surface area, proficiency of molecule exchange, and metabolic capacity in a wide range of branched systems such as plants (37), capillary veins, and inner mitochondrial membranes (26). Hence, cells in mature bacterial pellicles, by exploiting fractal wrinkle morphology, likely enjoy accelerated growth and metabolic and signal transduction benefits that are absent in smooth and nonfractally structured populations.

## The Memory of the Heterogeneous Microstructure Is Preserved during *V*. *cholerae* Pellicle Morphogenesis

**Fig. 4.**
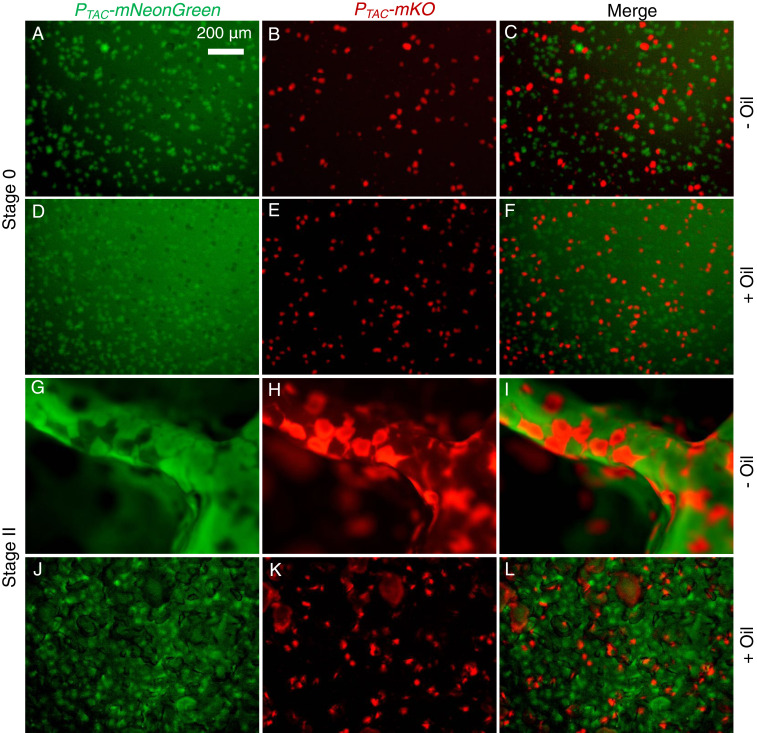
Individual *V*. *cholerae* microcolonies remain segregated, and the microstructural “memory” of the pellicle is preserved despite global morphological transitions. A 1:1 mixture of cells of two otherwise isogenic *V*. *cholerae* strains constitutively expressing either *mNeonGreen* (green) or *mKO* (red) was used to inoculate pellicles. The combined initial inoculum is OD_600_ = 0.01. (*A*–*F*) Top views of the distributions of *V*. *cholerae* pellicle microcolonies in Stage 0 for (*A*–*C*) a liquid–air interface (no mineral oil) and (*D*–*F*) a liquid–liquid interface (with mineral oil). The left column shows colonies expressing *P*_*TAC*_-*mNeonGreen*, the middle column shows colonies expressing *P*_*TAC*_-*mKO*, and the right column shows the merged images. (*G*–*L*) Top views of the distributions of *V*. *cholerae* pellicle microcolonies during Stage II secondary ridge instabilities for (*G*–*I*) at the liquid–air interface (no mineral oil) and (*J*–*L*) at the liquid–liquid interface (with mineral oil). (Scale bar, 200 μm.)

We explored the microstructural origin of the macroscopic *V*. *cholerae* pellicle morphology by inoculating pellicle formation chambers with mixed populations of isogenic *V*. *cholerae* cells labeled with two different fluorescent reporters. In the planar growth stage (Stage 0), the microcolonies of the two strains that formed at the fluid–fluid interface were segregated (Fig. 4 *A*–*F*). Since the differently labeled microcolonies touched one another, presumably due to the stochastic nature of the initial seeding, we wondered whether they mix and merge as the pellicle matures and expands. They do not: the microcolonies remained separated following the wrinkle and secondary morphological transitions (Fig. 4 *G*–*L*) producing a random polka-dot–like pattern in the three-dimensional (3D) pellicle surface. We note that the fluorescent reporter output is lower and cell growth is slower in pellicles grown under oil layers than in pellicles directly exposed to air due to reduced availability of oxygen to cells in the former. Consequently, the wrinkling amplitudes in the out-of-plane direction are different. In both cases, however, during pellicle morphogenesis, the memory of the initial microstructures in the flat planar stage is preserved throughout 3D maturation.

## The Dynamics of *V*. *cholerae* Pellicle Morphogenesis Are Controlled by Its Initial Microstructure

**Fig. 5.**
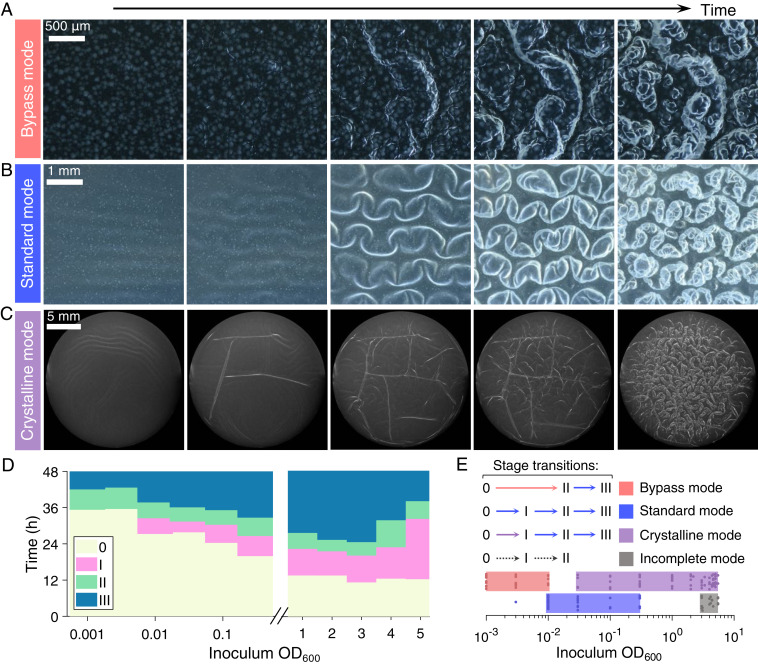
Dynamics of *V*. *cholerae* pellicle morphogenesis show distinct transition modes that are controlled by cell seeding density. (*A*–*C*) Top views of time-series images for three modes of pellicle morphological transitions, (*A*) the bypass mode in which primary wrinkling does not occur, inoculum OD_600_ = 0.001, from 30 to 49 h, (*B*) the standard mode in which stages 0, I, II, and III occur sequentially, inoculum OD_600_ = 0.01, from 21 to 49 h, and (*C*) the crystalline mode in which the pellicle partitions into discrete subdomains, inoculum OD_600_ = 3.35, from 7 to 25 h. (Scale bars, 500 μm, 1 mm, and 5 mm, respectively.) Images in *A* and *B* were acquired with a stereo microscope and in *C* with a single lens reflex camera to capture the full width of the chamber. (*D*) Time development of pellicle transition stages 0, I, II, and III for the designated inoculum seeding densities (*n* = 3 biological replicates for each seeding cell density). (*E*) The four distinct modes of pellicle morphogenesis are identified by the sequence and timing with which transitions occur. Pellicle morphological transition stages at the designated inoculum seeding densities are labeled. Data points denote pellicle biological replicates for technical triplicates.

We hypothesized that the initial pellicle microstructure formed by the founder microcolonies could determine the overall dynamics of pellicle morphogenesis. Since the wrinkling wavelengths of smooth uniform thin films vary with film thickness to the 3/4 power (25), local microcolony heterogeneity is expected to induce local variations in the strain and stress fields and, in so doing, alter the overall pellicle deformation profile, wrinkling wavelength (36), and, potentially, shape transition dynamics. To investigate these possibilities, we varied the initial *V*. *cholerae* seeding cell density, with inoculum OD_600_ values ranging from 0.001 to 4, to systematically reduce the founder colony size, colony density per surface area, and the thickness heterogeneity (*SI Appendix*, Fig. S3). In the standard mode, pellicle morphogenesis consists of Stage 0 planar growth of the founder cells and secondary layers, Stage I primary wrinkling, Stage II secondary ridge instability, and Stage III fractal wrinkling (Fig. 1). Time-course imaging of pellicle development starting from different cell seeding densities revealed three alternative sequences of morphogenic events. At low cell seeding densities (OD_600_ = 0.001 to 0.003), a direct transition from the flat plane to 3D ridge structures occurred, which was followed by the emergence of structures with fractal order. We call this sequence the bypass mode since primary wrinkling (Stage I) did not occur (Fig. 5*A* and Movie S3). Indeed, compared to the surface patterns that form in the standard mode (Fig. 5*B*), in the bypass mode, the pellicle develops larger microcolonies with less space between them prior to the onset of morphological transitions. Furthermore, the boundaries of the microcolonies establish local defects that pin the sites of formation of 2D ridges (*SI Appendix*, Fig. S6 and Movie S3), presumably by concentrating compressive strain similar to the bending at “soft spots” when the pellicle modulus heterogeneity is large (*SI Appendix*, Fig. S4*B*). The dramatic surface microcolony heterogeneity eliminates the occurrence of primary wrinkles. At higher cell seeding densities (OD_600_ = 0.05 to 3), another mode occurs in which primary wrinkles form and subsequently collide and undergo local wrinkle-to-fold transitions at defined linear boundaries (Fig. 5*C* and *SI Appendix*, Fig. S7). Subsequently, discrete crystalline domains emerge, and wrinkles within each domain become aligned and collectively transition to secondary ridges (Fig. 5*C* and Movie S4). We call this sequence the crystalline mode. Lastly, at very high cell seeding densities (OD_600_ ≥ 3), higher-order morphologies (Stage II or III) are not observed, and the pellicle remains a 2D sheet. We call this sequence the incomplete mode. The temporal dynamics of these modes could be captured by measuring the average onset times of Stages 0 through III (Fig. 5*D*). As cell seeding density increases, the morphological transitions occur earlier; however, above seeding densities of OD_600_ ∼ 2, the onset of primary wrinkling is delayed, and the ultimate morphologic stage achieved by the pellicle is limited (hence the term incomplete). The different dynamical transitions, as controlled by cell seeding densities, are summarized in a phase diagram (Fig. 5*E*). The emergence of the fractal wrinkles (Stage III) is quite general: it occurs for all seeding densities tested except the highest seeding density in which nutrients are insufficient to enable further pellicle progression. By contrast, the particular dynamics that lead to the fractal stage are controlled by the inoculum seeding densities.

## Conclusion

In this study, we report the morphological progression of *V*. *cholerae* pellicles at a fluid–fluid interface. As a model soft biomaterial, a bacterial pellicle consists of biocomponents and living cells that display active metabolism and growth. In contrast to the merging of wrinkles into folds that occur in the shape evolution of smooth passive films on a fluid bath, we find that bacterial pellicles undergo a hierarchy of morphological transitions culminating in a cascade of wrinkles with increasingly smaller wavelengths. The origins of the structural complexity and the fractal scaling in surface dimensionality reside in the founding bacterial microcolonies that form the basic unit of the pellicle. We linked the dynamics of macroscopic morphological transitions directly to the microcolony structures and showed that distinct developmental modes can proceed depending on the inoculum seeding density. The basic elements of bacterial pellicle morphogenesis, such as cell growth, matrix production, the accumulation of mechanical stresses, and morphogenic transformations are ubiquitous in both prokaryotic and eukaryotic multicellular systems. For example, in eukaryotes, the folding of the gut, the wrinkling of skin, and the fractal branching of capillary blood vessels share many of the structural and mechanical features we observe here for bacterial pellicles. By controlling the matrix constituents, nutrient acquisition, signaling gradients, mechanics, or flow perturbations, bacterial biofilms and pellicles could provide tractable model systems to understand the overarching principles underlying morphogenesis and for engineering of functional soft biomaterials.

## Materials and Methods

### Strains and Growth Medium.

*V*. *cholerae* strains used in this study are derivatives of the wild-type *V*. *cholerae* O1 biovar El Tor strain C6706 with a missense mutation in the *vpvC* gene (*vpvC*^W240R^, denoted Rg) that elevates cyclic diguanylate (c-di-GMP) levels (38, 39). This strain forms rugose (Rg) biofilms on solid agar plates. Because the Rg strain forms robust pellicles, it is used here as the parent strain for all pellicle assays. The strains used in this work are the following: *V*. *cholerae* BQ200A, *vpvC*^W240R^; *V*. *cholerae* BQ200D, *vpvC*^W240R^ ∆*vc1807*::*P*_*TAC*_-*mNeonGreen*-*Spec*^R^; and *V*. *cholerae* BQ200P, *vpvC*^W240R^ ∆*vc1807*::*P*_*TAC*_-*mKO*-*Spec*^R^. LB medium was used in all experiments.

### Pellicle Development.

*V*. *cholerae* strains were grown overnight at 37 °C in LB liquid medium with shaking. Cell clusters were dispersed by vortex for 1 min with 1 mm glass beads (Biospec). The resulting cell suspensions were back diluted 100-fold and incubated for an additional 2 h with shaking at 37 °C in LB medium so that the cultures reached early exponential phase (OD_600_ = 0.1-0.2). Following another 1 min of vortex with beads, cultures were diluted with LB medium to yield inoculum cell densities ranging from OD_600_ = 0.001 to 0.1. When higher inoculum seeding densities were necessary, the growth time following resuspension was increased accordingly. A total of 5.5 mL of culture inoculum was added to wells of 12-well plates so that the depth of liquid in each well was 14 mm. The cultures in the wells were overlaid with sterile mineral oil (Sigma-Aldrich, volume 850 μL) to prevent evaporation and pellicle desiccation. Pellicles were allowed to form at room temperature (20 to 22 °C) and imaged from 15 h to 48 h as specified.

### Stereo Microscopy Imaging.

Time-course images of pellicle development were acquired with a custom-built stereo microscope setup using a 2× plan apochromatic objective with numerical aperture 0.1. Sample scanning in the vertical direction was accomplished using a motorized micrometer stage controlled by MATLAB via a microcontroller. The vertical position of the pellicle at the fluid–fluid interface was continuously tracked using an auto-focusing algorithm based on grayscale local variances (40) in acquired image stacks. The vertical scan range for each subsequent acquisition was automatically adjusted to keep the central morphological features of the pellicle in focus. Images of fluorescent pellicles were taken with a Leica M205FA stereo microscope using a 1× plan apochromatic objective with numerical aperture 0.35.

### Image Processing.

Volumetric image stacks acquired using the custom stereo microscope were first merged into a single image using a focus stacking algorithm based on grayscale local variance (41). The sequence of focus-stacked images in each time-course experiment were next registered in the *x*-*y* plane using intensity-normalized cross-correlation methods. To extract wrinkle features, Frangi vesselness filtering (42, 43) was applied to the focus-stacked images with a filter length scale from 20 to 50 μm. The output ridge intensities were used for wavelength and curvature analyses.

### Fractal Dimensions of Pellicle Surfaces.

To quantify the fractal behavior and fractal dimensions of pellicle wrinkles, Frangi vesselness maps of pellicles were first binarized using intensity thresholding and then skeletonized using medial axis thinning algorithms (44). Short and isolated branches in the skeletons were pruned. A standard box-counting algorithm was applied to the skeleton images and the scaling exponents of the box counts to the box sizes were obtained to define the fractal dimensions (37, 45).

### Mathematical Modeling of the Primary Wavelength for a Heterogeneous Pellicle.

To model the material heterogeneity of a *V*. *cholerae* pellicle, we consider a 2D thin elastic film patterned in the *x*-direction with alternating hard and soft intervals, i.e., with high and low bending moduli. The hard intervals are intended to model the microcolonies (Fig. 2*E* and *SI Appendix*, Fig. S4). The film is assumed to be uniform in the *y*-direction. Specifically, the bending modulus is given by:

$$B\left(x\right)=\left\{ \begin{array}{lc} B_{\text{H}}\; & x_{0}+n\lambda_{B}<x\leq x_{0}+\left(n+1/2\right)\lambda_{B}\\ B_{\text{S}}\; & x_{0}+\left(n+1/2\right)\lambda_{B}<x\leq x_{0}+\left(n+1\right)\lambda_{B} \end{array}\right.\;\left(n\in \mathbb{Z}\right),$$ [1]

where $x_{0}$ is an arbitrary reference point and $\lambda_{B}$ is the wavelength of the alternating modulus pattern.

For each segment of homogeneous modulus $B_{i}\;\left(i=\text{H},\text{S}\right)$, the vertical displacement w of the modeled pellicle is determined by the Föppl–von Kármán equation:

$$B_{i}\frac{d^{4}w}{dx^{4}}+t\frac{d^{2}w}{dx^{2}}+\rho gw=0,$$ [2]

where t is the longitudinal compressive stress resulting from pellicle growth against the side walls in the experiment and ρg is the specific weight of the liquid on which the pellicle grows. To simplify the notation, we define a normalized x coordinate $\tilde{x}=k_{0}x$, where $k_{0}={\left(\frac{2\rho g}{B_{\text{H}}+B_{\text{S}}}\right)}^{1/4}$ denotes the wrinkling wavenumber of a film of uniform modulus $B_{0}=\frac{B_{\text{H}}+B_{\text{S}}}{2}$ and the dimensionless stress $\tilde{t}=\frac{t}{B_{0}k_{0}^{2}}$. The modulus patterning can be characterized by two dimensionless parameters: the amplitude $\beta_{B}\equiv \frac{B_{\text{H}}-B_{\text{S}}}{B_{\text{H}}+B_{\text{S}}}$ and the wavelength $\beta_{\lambda }\equiv \frac{k_{0}\lambda_{B}}{2\pi }$. Upon nondimensionalizing the Föppl–von Kármán Eq. **2**, we obtain:

$$\left(1\pm \beta_{B}\right)w^{''''}+\tilde{t}w^{''}+w=0,$$ [3]

where ' denotes the derivative with respect to $\tilde{x}$, and the hard and soft portions take the + and – signs, respectively. The general solution of Eq. **3** is given by:

$$w\left(\tilde{x}\right)=C_{i}^{\left(1\right)}\exp\left(q_{i}^{+}\tilde{x}\right)+\;C_{i}^{\left(2\right)}\exp\left(-q_{i}^{+}\tilde{x}\right)+\;C_{i}^{\left(3\right)}\exp\left(q_{i}^{-}\tilde{x}\right)+\;C_{i}^{\left(4\right)}\exp\left(-q_{i}^{-}\tilde{x}\right),$$ [4]

where i=H,S and ${\left(q_{i}^{\pm }\right)}^{2}$ are the two roots of the eigenequation $\left[1+\text{sgn}\left(i\right)\beta_{B}\right]q^{4}+\tilde{t}q^{2}+1=0$ in which sgn(H)=1 and sgn(S)=−1.

The boundary conditions at the interface ${\tilde{x}}_{*}$ between the hard and soft portions are given by:

$${w|}_{{\tilde{x}}_{*}^{+}}={w|}_{{\tilde{x}}_{*}^{-}},\;{w^{\prime }|}_{{\tilde{x}}_{*}^{+}}={w^{\prime }|}_{{\tilde{x}}_{*}^{-}},\;B{w^{\prime \prime }|}_{{\tilde{x}}_{*}^{+}}={Bw^{\prime \prime }|}_{{\tilde{x}}_{*}^{-}},\;\text{and}\;B{w^{\prime \prime \prime }|}_{{\tilde{x}}_{*}^{+}}={Bw^{\prime \prime \prime }|}_{{\tilde{x}}_{*}^{-}},$$ [5]

where the latter two equations impose the balance of torque and shear stress at the joining interfaces. Introducing Eq. **4** into Eq. **5**, one can rewrite the boundary conditions in matrix form as $M_{\text{H}}\left({\tilde{x}}_{*}\right)\cdot {\vec{C}}_{\text{H}}=M_{\text{S}}\left({\tilde{x}}_{*}\right)\cdot {\vec{C}}_{\text{S}}\equiv \vec{f}\left({\tilde{x}}_{*}\right)$, where $M_{i}\left({\tilde{x}}_{*}\right)\;\left(i=\text{H},\text{S}\right)$ is given by:

$$\{\begin{array}{c} M_{i}\left({\tilde{x}}_{*}\right)\equiv \left[v_{i}\left({\tilde{x}}_{*};q_{i}^{+}\right),v_{i}\left({\tilde{x}}_{*};-q_{i}^{+}\right),v_{i}\left({\tilde{x}}_{*};q_{i}^{-}\right),v_{i}\left({\tilde{x}}_{*};-q_{i}^{-}\right)\right]\\ v_{i}\left(x;q\right)=\exp\left(qx\right){\left[1,\;q,\;q^{2}\left(1+\text{sgn}\left(i\right)\beta_{B}\right),\;q^{3}\left(1+\text{sgn}\left(i\right)\beta_{B}\right)\right]}^{T} \end{array},$$ [6]

and ${\vec{C}}_{i}={\left[C_{i}^{\left(1\right)},C_{i}^{\left(2\right)},C_{i}^{\left(3\right)},C_{i}^{\left(4\right)}\right]}^{T}$.

Using the relations $\vec{f}\left({\tilde{x}}_{0}\right)=M_{\text{H}}\left({\tilde{x}}_{0}\right)\cdot {\vec{C}}_{\text{H}}$, $M_{\text{H}}\left({\tilde{x}}_{0}+\beta_{\lambda }/2\right)\cdot {\vec{C}}_{\text{H}}=M_{\text{S}}\left({\tilde{x}}_{0}+\beta_{\lambda }/2\right)\cdot {\vec{C}}_{\text{S}}$, and $\vec{f}\left({\tilde{x}}_{0}+\beta_{\lambda }\right)=M_{\text{S}}\left({\tilde{x}}_{0}+\beta_{\lambda }\right)\cdot {\vec{C}}_{\text{S}}$, we obtain:

$$\vec{f}\left({\tilde{x}}_{0}+\beta_{\lambda }\right)=M_{\text{S}}\left({\tilde{x}}_{0}+\beta_{\lambda }\right)M_{\text{S}}^{-1}\left({\tilde{x}}_{0}+\beta_{\lambda }/2\right)M_{\text{H}}\left({\tilde{x}}_{0}+\beta_{\lambda }/2\right)M_{\text{H}}^{-1}\left({\tilde{x}}_{0}\right)\cdot \vec{f}\left({\tilde{x}}_{0}\right)\equiv T\left(\beta_{\lambda }\right)\cdot \vec{f}\left({\tilde{x}}_{0}\right),$$ [7]

where we have defined the transfer matrix 
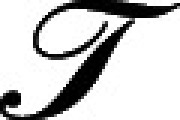
 and we have used the fact that 
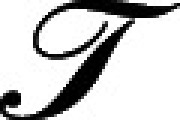
 is independent of ${\tilde{x}}_{0}$ due to the periodicity of the modulus pattern. When the dimensionless stress $\tilde{t}$ is small, for certain values of $\beta_{B}$ and $\beta_{\lambda }$, the eigenvalues have absolute values either larger or smaller than 1, and thus, the corresponding eigenmodes will either diverge or vanish at infinity. As $\tilde{t}$ increases, the primary wrinkling instability occurs when $\left|T\left(\beta_{\lambda }\right)-e^{ik^{*}\beta_{\lambda }}\right|=0$, which yields the wrinkle wavelength $\lambda^{*}=2\pi /k^{*}$ (Fig. 2).

### Finite Element Simulation of *V*. *cholerae* Pellicles.

We use the previously described framework of elastic growth (10, 11, 18) to model the primary wrinkling instability of *V*. *cholerae* pellicles. In brief, we use the deformation gradient tensor F=∂x/∂X to describe the local shape change of a 2D thin film, in which $x={\left[x,z\right]}^{T}$ and $X={\left[X,Z\right]}^{T}$ denote the current coordinates and the material coordinates, respectively. The overall shape change F can be decomposed into a contribution $F_{\text{g}}$ due to growth and a contribution $F_{\text{e}}$ due to elastic deformation (namely, $F=F_{\text{e}}F_{\text{g}}$). The planar growth is described by $F_{\text{g}}=\left[\begin{array}{cc} 1+\epsilon_{\text{g}} & 0\\ 0 & 1 \end{array}\right]$ with a growth-induced compressive strain $\epsilon =\epsilon_{\text{g}}/\left(1+\epsilon_{\text{g}}\right)$, where $\epsilon_{\text{g}}$ is the cumulative growth. Thus, the elastic deformation can be computed from $=FF_{\text{g}}^{-1}$. The Cauchy stresses σ associated with the elastic deformation $F_{\text{e}}$ are then computed from the elastic constitutive relation of the material. Here, we model the pellicle as an almost incompressible neo-Hookean elastic material (10, 11, 18) whose strain energy density in the material coordinate system is given by (46) $\Psi \left(F_{\text{e}}\right)=\frac{G}{2}\left(I_{\text{C}}-2-2\ln J\right)+\frac{G\upsilon }{1-2\upsilon }{\left(\ln J\right)}^{2}$, where G denotes the shear modulus, v≈0.5 denotes the Poisson’s ratio, $I_{\text{C}}\;=\;\text{tr}\left(F_{\text{e}}^{T}F_{\text{e}}\right)$ is the first invariant of the right Cauchy–Green deformation tensor, and $J\;=\;\text{det}\left(F_{\text{e}}\right)$. Note that to capture the material heterogeneity of the *V*. *cholerae* pellicle, the elastic modulus G=G(X) could vary with the material coordinates. The equilibrium configuration of the pellicle is obtained by solving the force balance equation:

∇⋅σ=0, [8]

with a boundary condition $\sigma_{n}=-\rho gz$ at the bottom surface of the film where ρg is the specific weight of the liquid on which the pellicle grows. A stress-free boundary condition is prescribed at the top surface of the film, and periodic boundary conditions are prescribed in the horizontal direction.

The numerical solutions of Eq. **8** were obtained by performing finite element simulations using the open-source computing platform FEniCS (47). The 2D simulation domain was discretized by second-order triangular elements using the Python package pygmsh (48), and the accuracy of the results was verified by mesh refinements. Eq. **8** was first rewritten in the Lagrangian frame of reference, and the weak form of the equation was obtained by taking variational derivatives using built-in functions in FEniCS. To ensure numerical convergence, we set Poisson’s ratio to be v=0.4, and we used a growth increment of $\Delta \epsilon_{\text{g}}\;=\;0.002$. For each step, a small random perturbation was first applied, and the force balance equation was solved with an adaptive dynamic relaxation scheme (49). In all simulations, the size of the simulation domain was set to be larger than 20 times the wavelength to minimize the finite size effect.

## Supplementary Material

Supplementary File

Supplementary File

Supplementary File

Supplementary File

Supplementary File

## Data Availability

All study data are included in the article and/or supporting information.

## Change History

May 14, 2021: Eq. 5, Eq. 7, and the main text have been updated.
