# Supplementary material for: Hierarchical transitions and fractal wrinkling drive bacterial pellicle morphogenesis
Source: Proc Natl Acad Sci U S A. 2021 May 10;118(20):e2023504118. doi: 10.1073/pnas.2023504118 (PMC8157956; doi:10.1073/pnas.2023504118)
Supplement: Supplementary File [file pnas.2023504118.sapp.pdf]

PNAS
www.pnas.org

Supplementary Information for
Hierarchical Transitions and Fractal Wrinkling Drive Bacterial Pellicle Morphogenesis

Boyang Qin, Chenyi Fei, Bruce Wang, Howard A. Stone, Ned S. Wingreen, Bonnie L. Bassler

Bonnie L. Bassler
Email: bbassler@princeton.edu

This PDF file includes:

Figures S1 to S8
Legends for Movies S1 to S4

Other supplementary materials for this manuscript include the following:

Movies S1 to S4

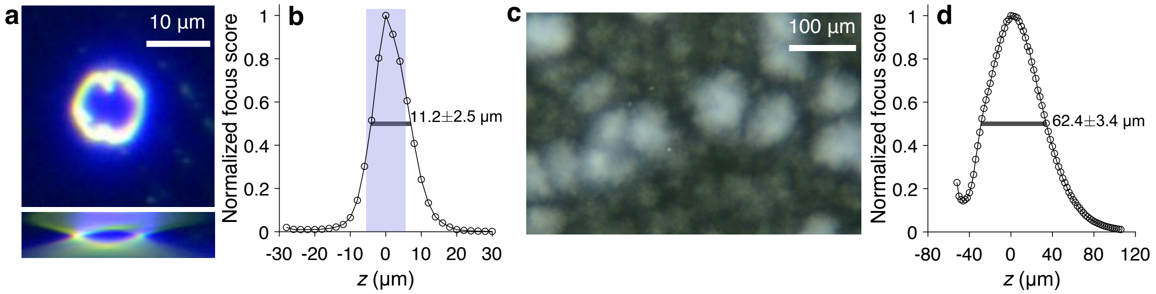

Fig. S1. Focus scoring calibration and pellicle thickness measurements. (**a**) Top: planar view of a calibration polystyrene bead (11 μm nominal diameter) imaged using custom stereoscopy. Bottom: orthogonal view in the *z* direction shows the volumetric scan where the particle sequentially goes in and out of focus. (**b**) The normalized focus score defined by gray level local variance for the calibration bead in (a). Shaded region (blue) indicates nominal prediction; black band indicates full width at half maximum. (**c**) Planar view of a *V. cholerae* pellicle from a seeding inoculum of $OD_{600} = 0.001$ and (**d**) the pellicle nominal thickness obtained from the full width at half maximum of the normalized focus score.

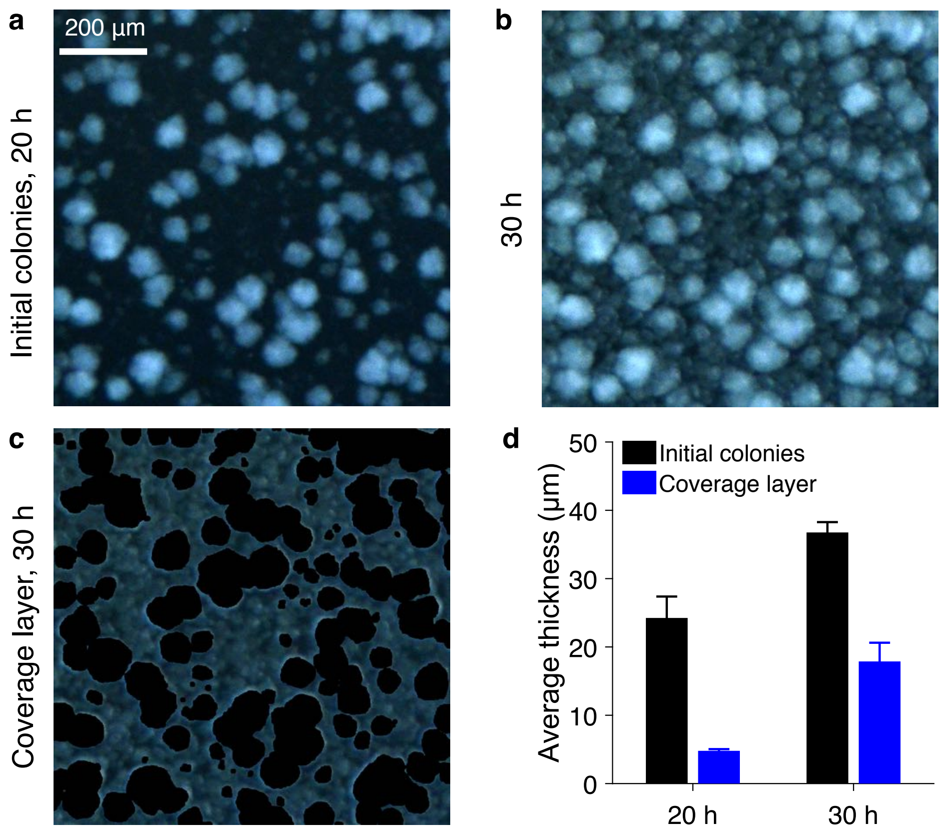

Fig. S2. The coverage layer, in addition to the founding microcolonies, emerges prior to confluence and the onset of primary wrinkling during *V. cholerae* pellicle development. (**a**) Top view of the initial founder layer of microcolonies at 20 h, with cell seeding density OD_{600} = 0.001. Empty void spaces are present between founder microcolonies. (**b**) Top view of the same location as in (a) at 30 h, which is immediately prior to the onset of morphological transitions. (**c**) Top view of the coverage layer at 30 h that filled the void spaces that had been present at 20 h. The image in (c) was acquired by first registering images (a) and (b) and, subsequently, subtracting the dilated (a) image from that of (b) to display only the layer of cells that was absent at 20 h. In the image, dilation was used to exclude the slight increases that occur in the radii of the founder colonies due to growth. (**d**) Comparison of the average thicknesses of the initial layer of founder microcolonies and the coverage layer at 20 h and 30 h showing the development of the coverage layer (n = 3 biological replicates). Intensity based thickness measurements with calibration via focus scoring were used. Error bars denote standard errors.

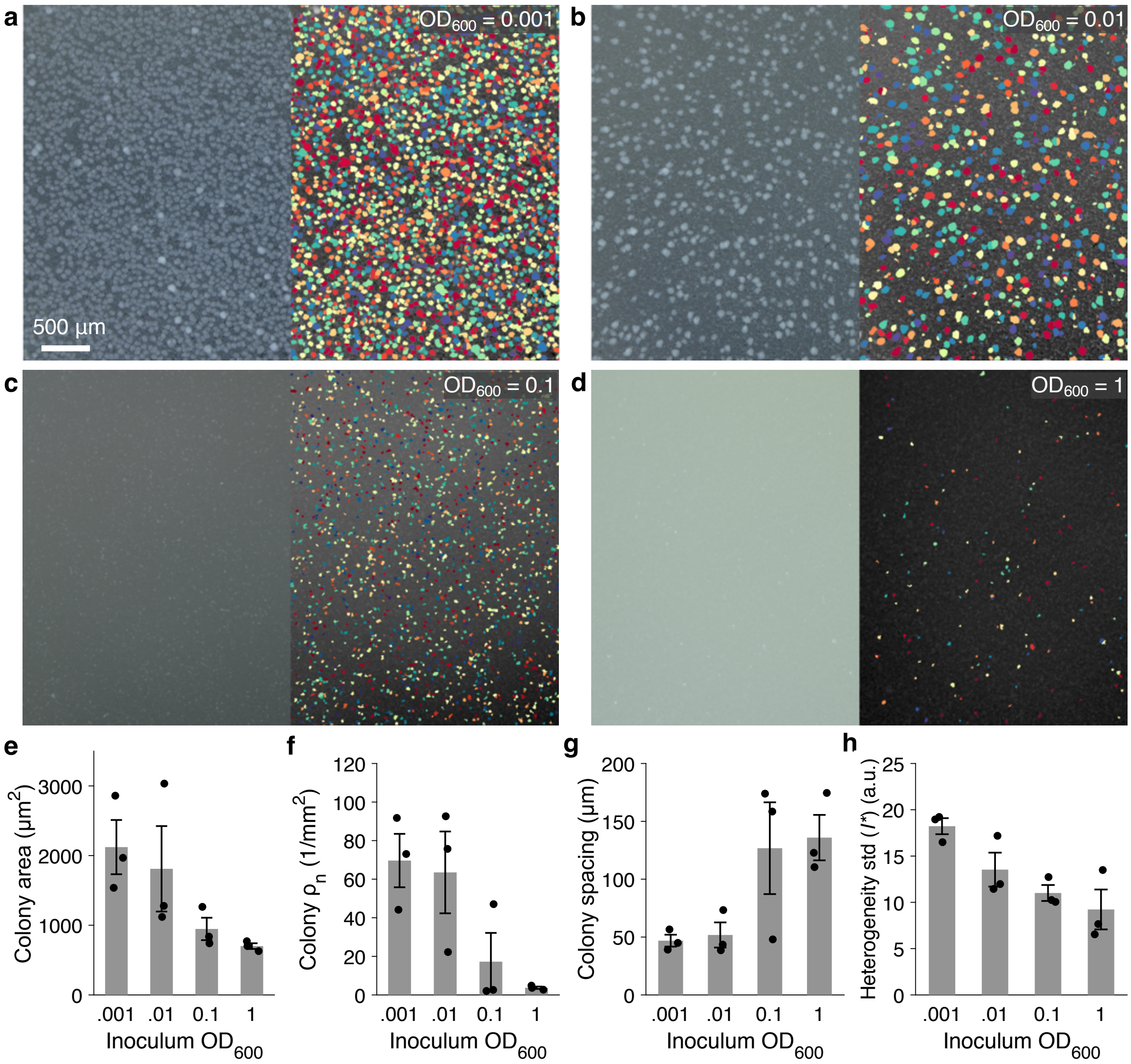

Fig. S3. *V. cholerae* pellicle microstructures prior to the onset of morphological transition are controlled by the initial seeding cell density. (**a-d**) Top view of pellicle microcolonies immediately prior to the onset of the first morphological transition for inoculum cell seeding density OD_{600} from 0.001 to 1, respectively. Left: focus projection of volumetric image stack. Right: founder microcolonies segmented by watershed algorithm. (**e**) Average founder colony size, as measured by segmented area. (**f**) Founder colony density per surface area. (**g**) Average spacing of microcolonies as measured by the distance to the nearest neighbor. (**h**) Pellicle apparent thickness heterogeneity as measured by the standard deviation of the reflected intensity I^*, corrected for spatial gradients in illumination. In (e-h), n = 3 biological replicates for each inoculum seeding cell density and error bars denote standard errors.

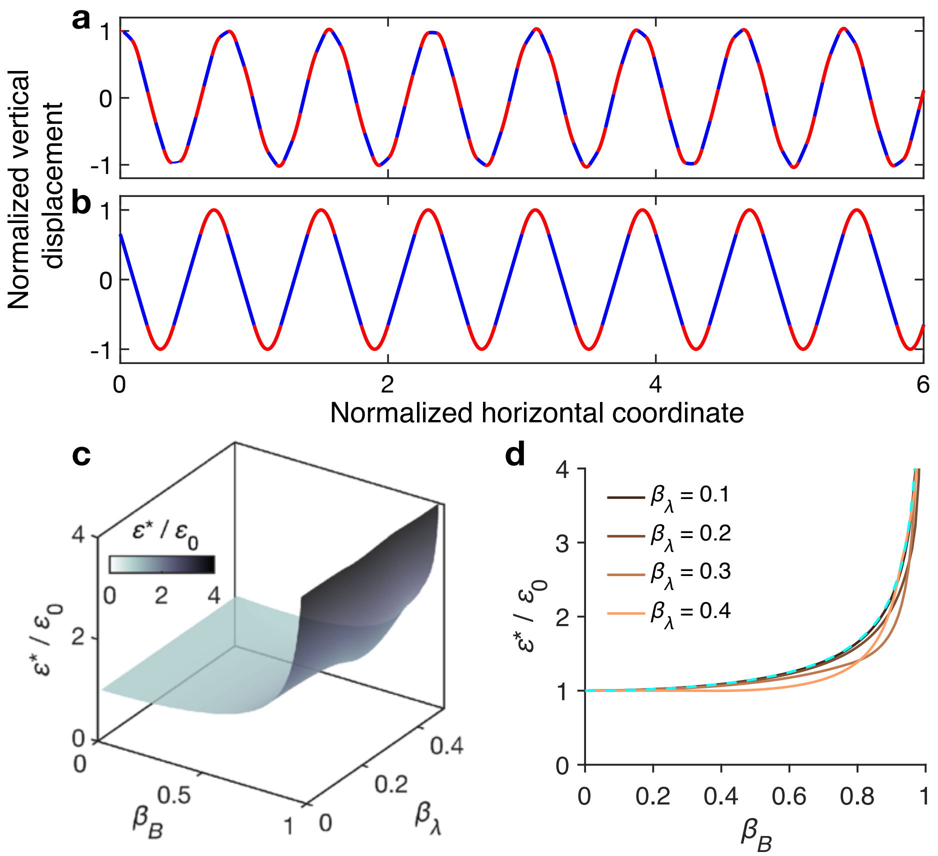

Fig. S4. An analytical model of *V. cholerae* pellicles possessing heterogenous moduli shows two wrinkling regimes and that the critical compressive strain for wrinkling is increased compared to that in smooth pellicles. (**a, b**) Normalized profiles of the two wrinkling modes. (**a**) For a heterogeneous pellicle with relatively fine microcolony features ($\beta_\lambda = 0.12$, $\beta_B = 0.8$), the wavelength of the sinusoidal wrinkle is locally reduced compared to that of a homogeneous pellicle. Both the horizontal coordinate and the vertical displacement are normalized by the uniform film wrinkling wavelength k_0. (**b**) For large microcolony features ($\beta_\lambda = 0.4$, $\beta_B = 0.8$), the soft regions are localized at the peaks and valleys while the hard regions remain essentially undeformed. (**c**) Dependence of the normalized critical compressive strain $\varepsilon^*/\varepsilon_0$ for the primary wrinkling on the modulus ratio β_B and length scale ratio β_λ. Here, ε^* is the critical compressive strain of the heterogeneous film and ε_0 is the critical compressive strain of a smooth film with a uniform bending modulus. (**d**) Representative cutlines through the isosurface in (c). Colors denote β_λ values. The dashed cyan curve represents the critical compression prediction from harmonic averaging of the pellicle moduli.

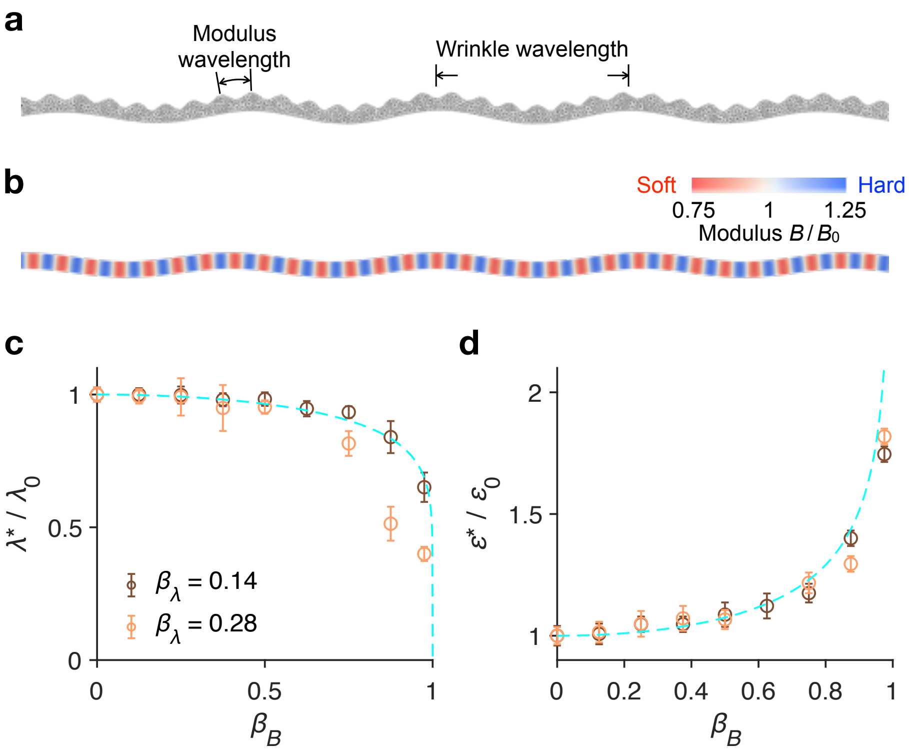

Fig. S5. Finite element simulation verifies the decrease in wrinkle wavelength and the increase in critical compressive strain observed in the analytical model. (**a, b**) Schematics of the wrinkling finite element simulations. The heterogenous bending moduli are modeled by (**a**) sinusoidally varying thicknesses, and (**b**) sinusoidally varying elastic moduli. Colors in (b) denote the normalized moduli. In (b), the modulus ratio is $\beta_B = 0.25$ and the length scale ratio is $\beta_\lambda = 0.14$. (**c**) Wrinkle wavelength and (**d**) critical compression are plotted against β_B for the finite element model in (b) at the designated β_λ values. The dashed cyan curves represent the predictions from harmonic averaging of the pellicle moduli.

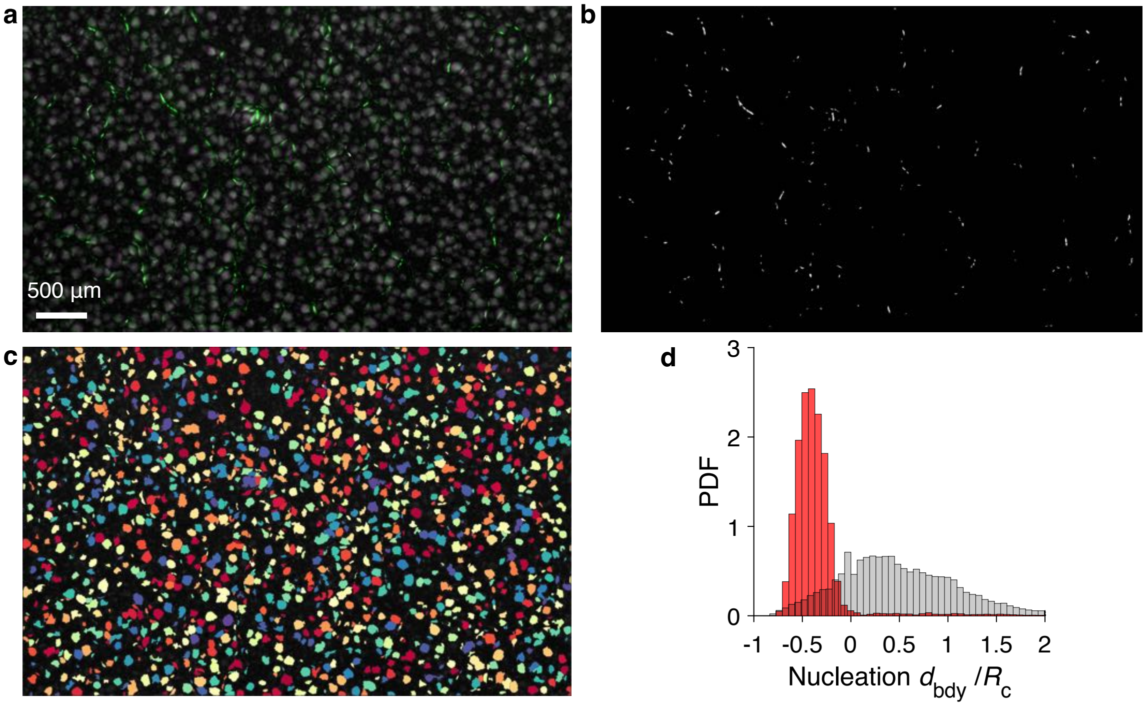

Fig. S6. Nucleation sites that designate the initiation of the bypass transition reside near the boundaries of the founder microcolonies. Nucleation sites are characterized by the onset of large local deformations and rapid changes in local image intensity. This property enabled the identification of the positions of the nucleation sites. Specifically, the locations of nucleation sites were pinpointed using image subtraction between the timepoint immediately following the bypass transition and the timepoint immediately prior to the transition. **(a)** Top view of a *V. cholerae* pellicle immediately after the bypass transition (35 h, green) overlaid on the microcolony structure immediately prior to the transition (34 h, magenta) for seeding cell density of OD_{600} = 0.001. Due to growth, microcolonies in the earlier timepoint overlap with those in the later timepoint and appear white in the merged image. By contrast, the nucleation sites where the bypass transition initiates are indicated by the strong green signals. (**b**) Spatial distribution of nucleation sites obtained by intensity subtraction and thresholding of the two images in (a). Both images were registered prior to subtraction using affine transformation. (**c**) Segmented microcolonies and the associated boundaries at the onset of the bypass transition. Colors denote distinct microcolonies. (**d**) The probability distribution function (PDF) of the distance between a nucleation site and the nearest microcolony boundary (d_{bdy}), normalized by the radius of that microcolony (R_c), shown in red. A negative value on the x-axis means that a nucleation site is within the microcolony boundary and a positive value indicates that the site is outside the microcolony perimeter. The distribution shown in gray depicts a control data set obtained by vertically inverting and randomly shifting the nucleation sites relative to the microcolonies. The distance ratio is spread across the possible values set by the microcolony spacing, a length scale similar to the colony size (Fig. S3e,g).

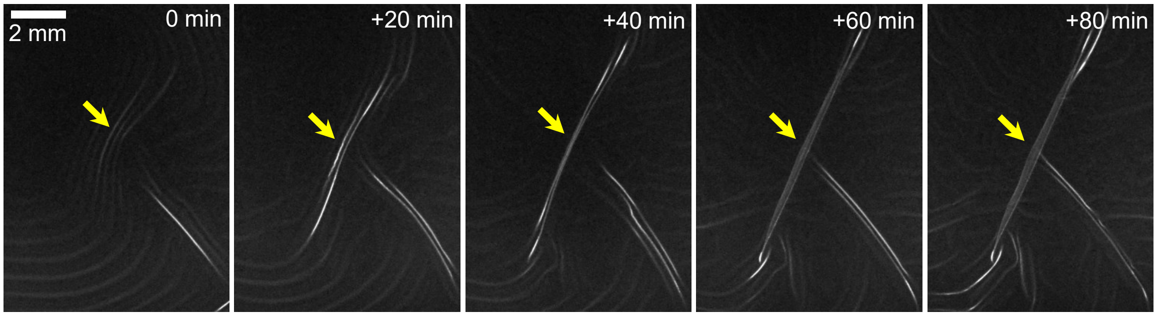

Fig. S7. A localized wrinkle-to-fold transition marks the pellicle crystalline boundaries. Time course imaging of *V. cholerae* pellicle morphology in the crystalline mode with initial cell seeding density of OD_{600} = 2. The periodic wrinkles localize and merge into an S-fold, which forms the boundaries of the sub-domains, as indicated by the arrows.

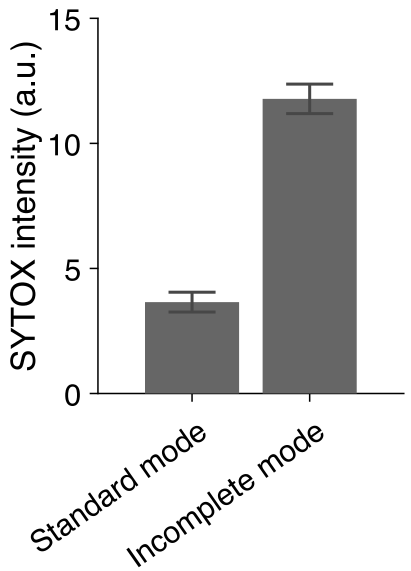

Fig. S8. Assessment of dead cells in *V. cholerae* pellicles. SYTOX fluorescent intensity staining of dead *V. cholerae* cells was quantified for pellicles in the standard morphogenic mode in which fractal wrinkling is present (inoculum $OD_{600} = 0.01$) and for pellicles in the incomplete morphogenic mode in which fractal wrinkling is absent (inoculum $OD_{600} = 3$) at 21 h post inoculation and at three separate locations focused on the top layer of the pellicles. Background autofluorescence was determined at the same 21 h time point in pellicles formed from identical cell seeding densities but to which no SYTOX stain was added. The background fluorescence was subtracted from the plotted intensity levels shown in the figure. In each case, the dye to cell ratio was maintained at 3 μM per OD_{600}. Error bars denote standard deviations from $n = 4$ biological replicates.

Supplementary Movie Captions

Movie S1. Volumetric stack of *Vibrio cholerae* pellicle morphogenesis at Stage III, fractal wrinkling. Step size in the vertical direction 50 μm. Inoculum cell density OD_{600} = 0.01.

Movie S2. Top view of the focus projection of the Standard mode of *Vibrio cholerae* pellicle morphogenesis at a liquid-liquid interface. Inoculum cell density OD_{600} = 0.01.

Movie S3. Top view of the focus projection of the Bypass mode of *Vibrio cholerae* pellicle morphogenesis at a liquid-liquid interface. Inoculum cell density OD_{600} = 0.001.

Movie S4. Top view of the focus projection of the Crystalline mode of *Vibrio cholerae* pellicle morphogenesis at a liquid-liquid interface. Inoculum cell density OD_{600} = 3.35.
